# microRNA-483 deficiency promotes loss of pancreatic β-cell identity and aberrant glucagon production under metabolic stress

**DOI:** 10.1210/endocr/bqag110

**Published:** 2026-09-26

**Authors:** Katy Matson, Aaron MacLeod, Ellie Sempek, Jordan Noble, Bryce Tapert, Farhad Alipour, Xiaohu Tang, Aimee H Marceau, Leslie S Satin, Xiaoqing Tang

**Affiliations:** Department of Biological Sciences, Michigan Technological University, Houghton, MI 49931, USA; Department of Biological Sciences, Michigan Technological University, Houghton, MI 49931, USA; Department of Biological Sciences, Michigan Technological University, Houghton, MI 49931, USA; Department of Biological Sciences, Michigan Technological University, Houghton, MI 49931, USA; Department of Biological Sciences, Michigan Technological University, Houghton, MI 49931, USA; Department of Biological Sciences, Michigan Technological University, Houghton, MI 49931, USA; Department of Biological Sciences, Michigan Technological University, Houghton, MI 49931, USA; Genomic Sequencing Lab, Michigan Technological University, Houghton, MI 49931, USA; Department of Pharmacology, Brehm Center for Diabetes, University of Michigan, Ann Arbor, MI 48109, USA; Department of Biological Sciences, Michigan Technological University, Houghton, MI 49931, USA

**Keywords:** islets, microRNA, insulin, glucagon, Aldh1a3, dedifferentiation

## Abstract

Pancreatic β-cell dedifferentiation is a pathological mechanism that contributes to β-cell dysfunction and loss during type 2 diabetes, but the underlying mechanisms remain incompletely understood. MicroRNAs (miRNAs) are key post-transcriptional regulators of cellular identity and function. We previously identified miR-483 as a β-cell–enriched miRNA that promotes β-cell function by targeting Aldh1a3. Here, we investigated its role under high-fat diet (HFD)/multiple low-dose streptozotocin (STZ)-induced metabolic stress. β-Cell–specific miR-483 deficiency exacerbated hyperglycemia and impaired glucose tolerance, accompanied by increased ALDH1A3 expression and reduced insulin expression in green fluorescent protein (GFP)-labeled cells. Under HFD/STZ-induced metabolic stress, miR-483 deficiency also increased glucagon production and the abundance of GFP^+^GCG^+^ and ALDH1A3^+^GCG^+^ cells, consistent with loss of β-cell identity and emergence of α-cell–like characteristics. Single-cell RNA sequencing revealed increased β-cells heterogeneity, including a subpopulation with glucagon expression and an altered endocrine phenotype. In addition, miR-483 deficiency was associated with mitochondrial fragmentation, impaired respiratory function, and increased oxidative stress. Together, these findings suggest that miR-483–ALDH1A3 axis contributes to the coordination of β-cell identity with mitochondrial and metabolic homeostasis during metabolic stress.

Progressive β-cell dysfunction is a central feature of the development and progression of type 2 diabetes (T2D) (1). Beyond apoptosis, accumulating evidence indicates that a substantial portion of β-cell failure also results from dedifferentiation, a process in which mature β-cells lose key transcriptional features of their differentiated state and acquire progenitor-like or alternative endocrine characteristics (2-4). Dedifferentiated β-cells exhibit reduced insulin production, re-expression of normally repressed “disallowed” genes, and increased susceptibility to metabolic and inflammatory stress (5, 6). Thus, identifying the molecular pathways that preserve β-cell identity and function under metabolic stress is essential for understanding β-cell failure and developing strategies to preserve β-cell function in diabetes.

Aldehyde dehydrogenase 1A3 (ALDH1A3) has emerged as a robust and conserved marker of β-cell dedifferentiation in both mouse and human diabetes (7-9). Increased ALDH1A3 expression is associated with impaired insulin secretion, oxidative stress, and metabolic dysfunction (9-11). However, the upstream mechanisms that regulate ALDH1A3 induction during β-cell dysfunction remain incompletely understood.

MicroRNAs (miRNAs) are important post-transcriptional regulators of β-cell identity, survival, and adaptive responses to metabolic stress (12). Several β-cell–enriched miRNAs, including miR-375 and miR-7, are dysregulated in diabetes, whereas disruption of global miRNA processing through Dicer deletion promotes β-cell dysfunction and dedifferentiation (13-16). miR-483, encoded within the Igf2 locus, is highly expressed in β-cells and has been implicated in endocrine lineage specification and metabolic regulation (17-19). We previously demonstrated that miR-483 deletion causes hyperglycemia, impaired glucose tolerance, and reduced insulin secretion under high-fat diet (HFD) stress while producing no overt metabolic phenotype under basal conditions (20). Interestingly, Aldh1a3 was validated as a direct target of miR-483, and elevated ALDH1A3 level was observed in HFD-induced miR-483 knockout mice (20). These findings suggest that miR-483 may contribute to the maintenance of β-cell identity, at least in part through regulation of ALDH1A3.

In the present study, we generated β-cell–specific miR-483 knockout mice with green fluorescent protein (GFP) lineage tracing and subjected them to combined HFD and low-dose streptozotocin (STZ) treatment to induce metabolic stress. We found that miR-483 deficiency markedly increased ALDH1A3

expression, altered β-cell heterogeneity, and was associated with the acquisition of α-cell–like features. miR-483 deficiency was also accompanied by increased glucagon production and impaired mitochondrial morphology and respiratory function. Together, these findings identify miR-483 as an important regulator of β-cell identity and metabolic adaptation and support a role for the miR-483–ALDH1A3 axis in protecting β-cell from stress-induced dysfunction.

## Materials and methods

### Generation of β-cell–specific miR-483 knockout mice with YFP lineage tracing

The generation and validation of the β-cell–specific miR-483 knockout mice (Ins1-Cre;miR483^fl/fl^) have been described previously (20). To generated β-cell–specific miR-483 knockout mice carrying a yellow fluorescent protein (YFP) lineage tracer, Ins1-Cre; miR483^fl/fl^ mice were crossed with Rosa26-YFP reporter mice (Stock No. 006148, the Jackson Laboratory). The resulting homozygous Ins1-Cre;Rosa-YFP;miR483^fl/fl^ male mice, referred to as miR483^−^/^−^, were used for the study. Ins1-Cre;Rosa-YFP male mice with the wild-type miR483 allele served as control mice (miR483^+/+^). Genotyping was performed on genomic DNA isolated from tail biopsies using PCR with primer sets listed in Table 1. All mice were housed in a specific pathogen-free facility at Michigan Technological University under a 12-hour light/12-hour dark cycle. Mice were fed standard chow or a HFD (60% kcal from fat, D12492, Research Diets) for 12 weeks and subsequently treated with multiple low-dose STZ (50 mg/kg/day for 5 consecutive days). Blood glucose levels were measured following a 6-hour fast, and plasma samples were collected on days 0, 7, 14, and 21 after STZ administration. Food and water were provided ad libitum. All procedures were approved by the Institutional Animal Care and Use Committee at Michigan Technological University.

**Table 1:** PCR primers used for mouse genotyping

Mice		Primers	Product size (bp)
miR-483 knockout mice	Forward	TGGATGACTATCCTTGCTGG	339
	Reverse	GAGGCACCCAAAAACCACTCC	
Ins1-Cre mice	Forward	AAACGTTGATGCCGGTGAACG	500
	Reverse	GGTCTTGTAGTTGCCGTCGT	
Rosa26-YFP knock-in mice	Forward	AGGGCGAGGAGCTGTTCA	384
	Reverse	TGAAGTCGATGCCCTTCAG	
Rosa26-YFP wild-type mice	Forward	CTGGCTTCTGAGGACCG	142
	Reverse	TCAGGACAACGCCCACACA	

Islets were isolated and purified by intraductal perfusion of collagenase V (0.6 mg/mL) (Sigma) following the protocol described (21). The purified islets were cultured in RPMI 1640 media supplemented with 10% fetal bovine serum and 1% penicillin–streptomycin (Thermo Fisher) at 37 °C with 5% CO_2_ for 24 to 72 hours according to the experiments.

### Single-cell RNA sequencing and pathway enrichment analysis

Approximately 200 islets from 2 control (miR483^+/+^) and 2 knockout (miR483^−/−^) mice were dissociated into single-cell suspensions using Accutase (Innovative Cell Technologies, #AT104) according to the manufacturer's instructions, and cell viability was assessed (22). Samples with >80% viable cells were submitted to the Michigan Technological University Genomic Sequencing Core for single-cell RNA sequencing (scRNA-seq). Single-cell capture, cDNA synthesis, and library preparation were performed using the 10× Genomics Chromium Single Cell 3′ v4 kit. Sequencing libraries were generated and managed through Illumina BaseSpace. Raw FASTQ files were processed using Cell Ranger v9.0.0 for read trimming, alignment, and gene counting. The GRCm39 mouse reference genome was customized using the Cell Ranger mkref function to incorporate the GFP reporter sequence. Downstream analyses were performed in R (v4.5.2) using Seurat v5 (23). Low-quality cells were removed based on unique molecular identifier counts, gene counts, and mitochondrial transcript percentage. Control and knockout datasets were integrated using the standard Seurat integration workflow.

Dimensionality reduction and visualization were performed using Uniform Manifold Approximation and Projection (UMAP), and cell clusters were annotated using established marker genes. Gene Ontology (GO) and Kyoto Encyclopedia of Genes and Genomes (KEGG) pathway enrichment analysis were performed on differentially expressed genes (DEGs) between miR483^+/+^ and miR483^−/−^ using clusterProfiler v.4.2.0 (24). Data visualization was generated in R using the ggplot2 v.4.0.3 and ComplexHeatMap v.2.26.1 packages (25, 26). The complete lists of DEGs are included in supplemental Datasets (27).

### Blood glucose, plasma insulin/glucagon measurements, and glucose tolerance test

Body weight and blood glucose levels were monitored weekly. For plasma hormone analysis, blood was collected from the postorbital vein using heparinized Natelson tubes (Fisher Scientific) and centrifuged at 1500 × g for 10 minutes at 4 °C to isolate plasma. Plasma insulin and glucagon levels were measured using the Ultrasensitive Mouse Insulin ELISA Kit (Crystal Chem Cat# 62100, RRID:AB_3662574) and Mouse Glucagon ELISA Kit (Crystal Chem Cat# 81518, RRID:AB_2811007), respectively.

Glucose tolerance tests (GTTs) were performed in mice as described (20). Mice were fasted for 16 hours and intraperitoneally injected with glucose at 1.0 g/kg body weight. Blood glucose measurements were taken from the tail vein at 0, 15, 30, 60, 90, and 120 minutes after injection. Blood glucose levels were plotted against time, and the area under the curve (AUC) was calculated. To measure plasma insulin/glucagon secretion in vivo, blood samples were collected from the postorbital vein at 0 and 15 minutes after glucose injection. To measure islet insulin/glucagon content, islets were snap frozen after harvest and lysed in passive lysis buffer. Islet insulin/glucagon contents were normalized to total protein levels.

### Immunofluorescence staining

Paraffin-embedded mouse pancreatic tissue sections were processed for immunostaining as described previously (20). Briefly, sections were deparaffinized, subjected to antigen retrieval, and incubated with indicated primary antibodies (listed in Table 2) overnight at 4 °C, followed by incubation with appropriate Alexa Fluor (Invitrogen) secondary antibodies (1:500) at room temperature for 2 hours. Notably, the anti-GFP antibody cross-reacts with YFP. Slides were counterstained with DAPI (4′,6-diamidino-2-phenylindole) (Sigma) and mounted using antifade mounting medium (Vector Labs). Images were acquired by using Olympus FluoView FV1000 confocal microscope or Leica whole-slide fluorescence scanner. Islet area was quantified using Image-Pro image analysis software. For all immunostaining analyses, a minimum of 4 animals and 5 tissue sections per animal (spaced 100 μm apart) were evaluated.

**Table 2:** Primary antibodies for immunostaining

Antigen	Host species	Dilution	Catalog no./identifiers	Company
Insulin	Mouse	1:500	I2018; RRID:AB_260137	Sigma
Insulin	Guinea pig	1:200	PA126938; RRID:AB_794668	Invitrogen
Glucagon	Mouse	1:200	G2654; RRID:AB_259852	Sigma
Glucagon	Rabbit	1:200	SAB4501137; RRID:AB_10761583	Sigma
GFP	Chicken	1:500	ab13970; RRID:AB_300798	Abcam
Aldh1a3	Rabbit	1:250	NBP2-15339; RRID:AB_2665496	Novus
MafB	Rabbit	1:100	NBP1-81342; RRID:AB_11005476	Novus
TOM20	Mouse	1:200	ab56783; RRID:AB_945896	Abcam

### Real-time RT-qPCR and Western blotting

RNA was extracted from isolated islets using the miRNeasy Kit (Qiagen) and treated with DNase I (Qiagen). cDNA was synthesized using the High-Capacity cDNA Reverse Transcription Kit (Thermo Fisher). Gene expression levels were detected with TaqMan assays (Applied Biosystems) and normalized to actin.

Proteins from cell lysates were separated by SDS-PAGE and analyzed by Western blot using anti-Aldh1a3 (Novus, Cat# NBP2-15339, RRID:AB_2665496) and anti-β-actin (Sigma, Cat# A5441, RRID:AB_476744) antibodies. Horseradish peroxidase–conjugated secondary antibodies (GE Healthcare) were used, and immunoblots were developed by using enhanced chemiluminescence (ECL) reagents (SuperSignal West Pico; Pierce).

### Adenoviral miRNA transduction

Recombinant adenoviruses expressing the miR-483 stem-loop precursor (Ad-miR483) were generated as previously described (20). An adenovirus expressing GFP alone (Ad-GFP) served as the control. Islets were dissociated with Accutase and infected with Ad-miR483 or Ad-GFP for 18 hours. Following infection, islets were washed twice with PBS and cultured for an additional 24 hours in a medium containing 0.5 mM palmitate, as previously described (20).

### Mitochondrial respiration and ATP production measurement

Oxygen consumption rate (OCR) was measured using a XFp Seahorse Analyzer (Seahorse Bioscience, MA, USA). Dispersed islets (20 per well) were preincubated for 4 hours in an assay medium containing 0.2% BSA and 2.8 mM glucose. Mitochondrial OCR parameters were assessed by sequential injections of 20 mM glucose, 1.5 μM oligomycin, 1 μM carbonyl cyanide-p-trifluoromethoxyphenylhydrazone (FCCP), and 0.5 μM antimycin A plus 0.5 μM rotenone (final concentrations). Total, glycolytic, and mitochondrial ATP production rates were measured using the Seahorse Real-Time ATP Rate Assay, following the sequential addition of 20 mM glucose, 1.5 μM oligomycin, and 0.5 μM antimycin A plus 0.5 μM rotenone. Each measurement cycle was performed in triplicate to obtain average values. Basal respiration, maximal respiration, and ATP production were calculated according to the manufacturer's guidelines.

### Intracellular reactive oxygen species measurement

Cytosolic reactive oxygen species (ROS) levels were assessed using 2′,7′-dichlorofluorescein diacetate (DCFH-DA), which is converted to fluorescent DCF upon oxidation by ROS. Isolated islets were washed with PBS and plated at 20 islets per well in a 96-well plate. Each well was incubated with 5 μM DCFH-DA in phenol-free DMEM for 60 minutes at 37 °C, followed by three PBS washes. Fluorescence was measured using an ELISA fluorimeter (excitation 488 nm, emission 535 nm) and normalized to total protein content per well.

### Statistical analysis

Data are presented as mean ± SEM. Statistical analyses were performed using GraphPad Prism 6. Significance was determined by unpaired Student's *t*-test or 1-way ANOVA, with *P* ≤ .05 considered statistically significant (*P < .05; **P < .01; ***P < .001).

## Results

### miR-483 deletion increases blood glucose, impairs glucose tolerance, and reduces insulin contents under high-fat diet/streptozotocin-induced metabolic stress

To investigate how miR-483 affects β-cell identity and function, we generated β-cell–specific miR-483 knockout mice carrying a YFP lineage tracer (Ins1-Cre; miR483^fl/fl^; Rosa-YFP, referred to as miR483^−^/^−^). Immunostaining confirmed that ∼90% of insulin^+^ β-cells expressed GFP/YFP (the anti-GFP antibody cross-reacts with YFP), whereas glucagon^+^ α-cells rarely exhibited GFP labeling under normal diet conditions (data not shown). Thus, we established GFP-labeled β-cell–specific miR-483 knockout mice (miR483^−^/^−^) and corresponding YFP control mice (miR483^+^/^+^) for lineage-tracing studies.

We previously demonstrated that when fed a chow diet, there were no difference in physiological parameters, including body weight, blood glucose, and plasma insulin between miR483^−^/^−^ and control miR483^+^/^+^ mice. We also reported that deletion of miR-483 increased blood glucose and impaired glucose tolerance, accompanied by reduced plasma insulin levels in mice after 20 weeks of HFD feeding (20). Consistently with these findings, following 12 weeks of HFD feeding and subsequent administration of multiple low-dose STZ injections (50 mg/kg for 5 consecutive days), miR483^−^/^−^ mice exhibited increased blood glucose levels and reached a peak at day 14 post-STZ compared with control miR483^+^/^+^ mice (Fig. 1A and 1B). Plasma insulin levels were significantly lower in miR483^−^/^−^ mice on days 0, 7, 14, and 21 post-STZ following a 6-hour fast (Fig. 1C). A GTT performed on day 10 post-STZ further demonstrated impaired glucose tolerance in miR483^−^/^−^ mice, which maintained higher blood glucose levels throughout the 2-hour period following glucose administration compared with miR483^+^/^+^ controls (Fig. 1D). These findings are consistent with the impaired glucose tolerance observed in miR483^−^/^−^ mice following HFD feeding alone (20).

**Figure 1:**
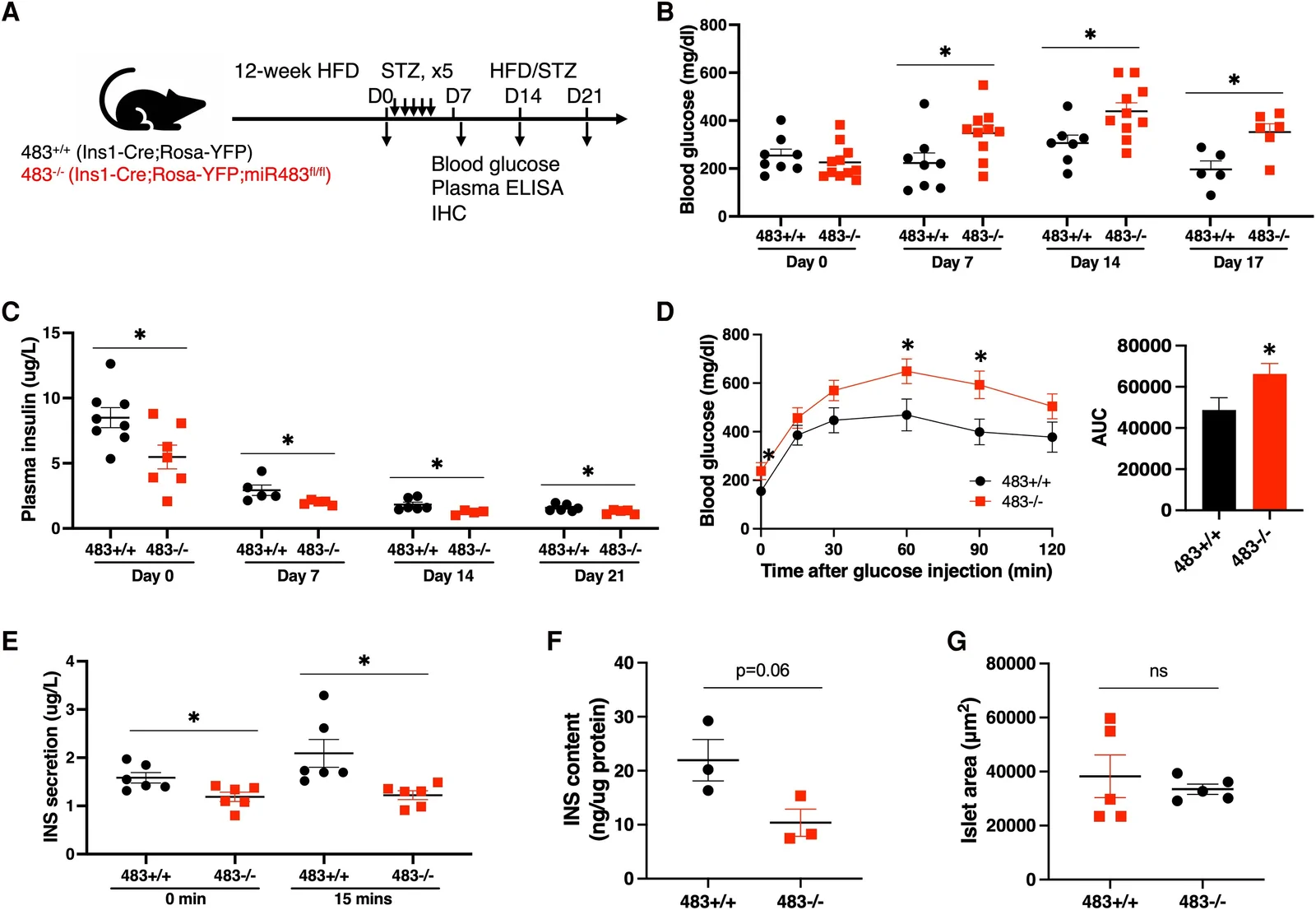
miR-483 deficiency increase blood glucose and reduces insulin secretion and islet insulin content under HFD/STZ-induced metabolic stress. (A) Schematic of the experimental design. Mice were fed a 12-week HFD followed by STZ administration (50 mg/kg for 5 consecutive days). (B) Blood glucose levels were significantly higher in miR483^−^/^−^ than in miR483^+^/^+^ control mice on days 7, 14, and 21 after STZ treatment. (C) Plasma insulin levels were correspondingly reduced in miR483^−^/^−^ mice. Blood glucose and plasma insulin were measured after a 6-hour fast (n = 5-12 per group). (D) Glucose tolerance test (GTT) was performed in male mice on day 10 post-STZ. Following a 16-hour fast, mice were intraperitoneally injected with glucose (1 g/kg body weight) and blood glucose level was measured at the indicated times. The area under the curve (AUC) was calculated to quantify glucose tolerance (n = 9-12 per group). (E) After a 6-hour fast, plasma insulin levels were measured at baseline (0 minutes) and 15 minutes after intraperitoneal glucose administration (1 g/kg body weight) (n = 6-7 per group). (F) Insulin content was measured in isolated islets and normalized to total protein content (n = 3 per group). (G) Islet area was quantified using Image-Pro image analysis software (n = 5 per group). All data were presented as means ± SEM. *P < .05 vs control.

Consistent with reduced fasting insulin levels, miR483^−^/^−^ mice also exhibited significantly lower plasma insulin levels following glucose administration (Fig. 1E), indicating impaired glucose-stimulated insulin secretion in vivo. In addition, miR-483 deficiency showed a trend toward reduced insulin content in isolated islets on day 15 post-STZ, although this difference did not reach statistical significance (*P* = .06; Fig. 1F). Despite this trend, islet area did not differ significantly between miR483^−^/^−^ and miR483^+^/^+^ mice (Fig. 1G), suggesting that the reduced islet insulin content was not attributable to gross changes in islet size.

### Lineage tracing reveals increased ALDH1A3 expression and loss of β-cell identity in miR-483 knockout mice under high-fat diet/streptozotocin-induced metabolic stress

We previously identified Aldh1a3 as a direct target of miR-483, with its expression elevated in miR483^−^/^−^ mice after 14 to 20 weeks of HFD (20). To determine whether increased ALDH1A3 expression was associated with the progressive loss of β-cell identity under metabolic stress, we examined ALDH1A3 expression in islets at different time points following STZ treatment. In line with this, ALDH1A3^+^ cells progressively increased in miR483^−^/^−^ islets on days 9, 15, and 21, reaching its highest level on day 15 compared with miR483^+^/^+^ controls (Fig. 2A and 2B). Lineage-tracing analysis showed that ALDH1A3^+^ cells were frequently detected within the GFP^+^ population, indicating their association with the insulin-expressing cell lineage. Notably, ALDH1A3^+^ cells often exhibited markedly reduced or undetectable insulin expression (indicated by white circles). The increased abundance of ALDH1A3^+^ cells were accompanied by less intense insulin staining in miR483^−^/^−^ islets compared with miR483^+^/^+^ controls, consistent with reduced β-cell identity. The proportion of ALDH1A3^+^INS^−^ cells was increased in miR483^−^/^−^ islets compared with miR483^+^/^+^ controls (approximately 16% vs 10%, respectively; Fig. 2C). Consistent with this finding, quantitative analysis of the GFP^+^ population revealed a higher proportion of GFP^+^INS^−^ cells in miR483^−^/^−^ islets than in miR483^+^/^+^ controls (20% vs 11%, respectively; Fig. 2D). Together, these findings indicate that miR-483 deficiency is associated with increased ALDH1A3 expression and reduced insulin expression among GFP-labeled cells under metabolic stress, supporting a role for the miR-483–ALDH1A3 axis in maintaining β-cell identity.

**Figure 2:**
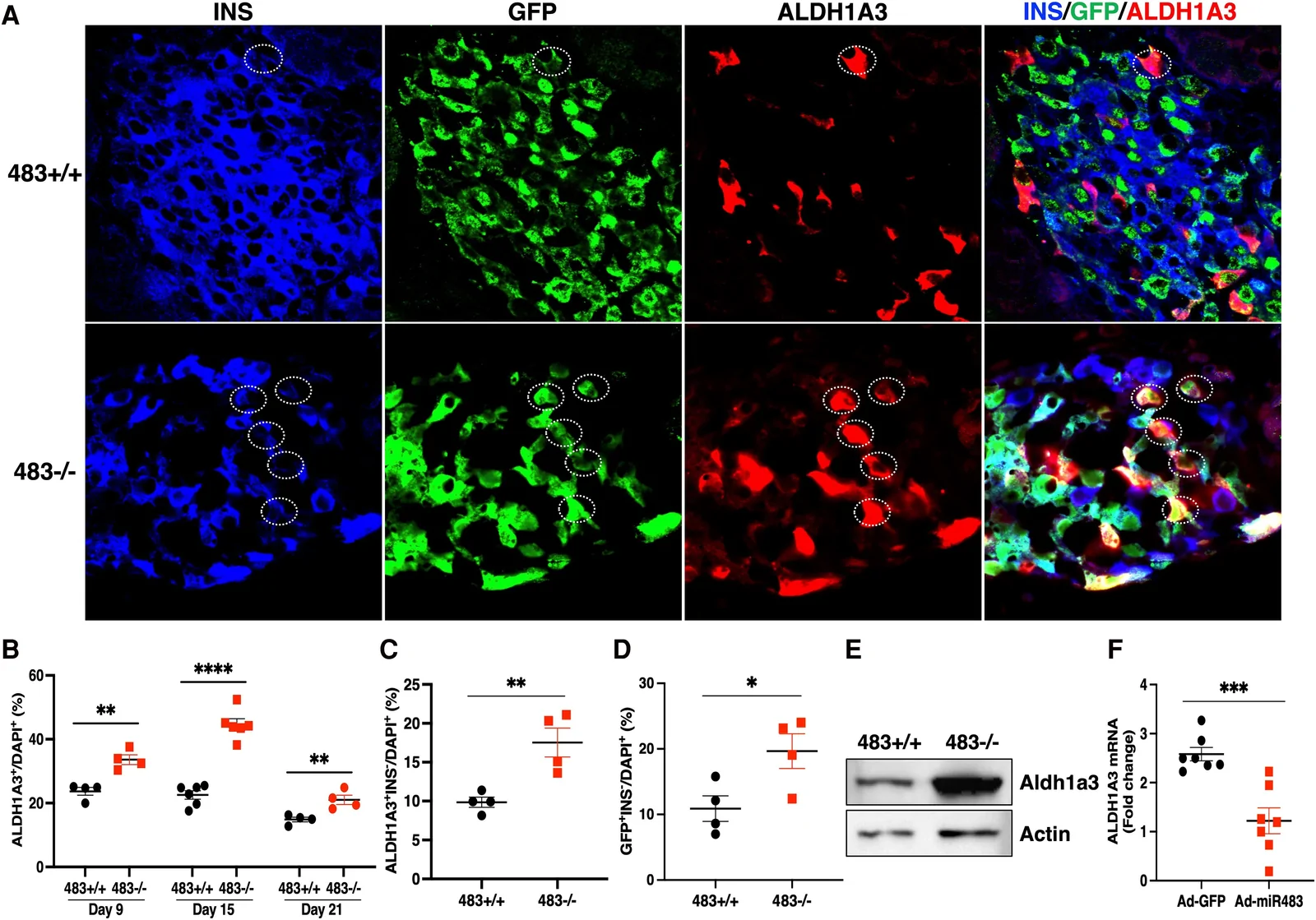
Lineage tracing reveals increased ALDH1A3 expression and loss of β-cell identity in miR-483 knockout mice under HFD/STZ-induced metabolic stress. (A) Immunofluorescence staining of pancreatic sections showing insulin (INS), GFP, and ALDH1A3. GFP^+^ALDH1A3^+^ cells with low or undetectable insulin expression (GFP^+^ALDH1A3^+^INS^−^) are indicated by white circles. (B-D) Quantification of total ALDH1A3^+^ cells (B), ALDH1A3^+^INS^−^ cells (C), and GFP^+^INS^−^ cells (D) in miR483^−^/^−^ and miR483^+^/^+^ mice. (E) Western blot analysis confirming elevated ALDH1A3 protein levels in islets isolated from miR483^−^/^−^ compared with miR483^+^/^+^ control mice. (F) Adenoviral overexpression of miR-483 (Ad-miR483) in isolated miR483^−^/^−^ islets significantly reduced ALDH1A3 level compared with Ad-GFP controls. Data are mean ± SEM (n = 4-8). *P < .05, **P < .01, ***P < .001, ****P < .0001 vs miR483^+^/^+^.

Western blot analysis confirmed that ALDH1A3 protein levels were markedly elevated in miR483^−^/^−^ islets compared with controls (Fig. 2E). Conversely, adenoviral overexpression of miR-483 (Ad-miR483) in isolated miR483^−^/^−^ islets significantly reduced ALDH1A3 expression compared with the Ad-GFP control (Fig. 2F). These findings are consistent with our previous identification of Aldh1a3 as a validated direct target of miR-483 and further support the miR-483–ALDH1A3 regulatory axis in pancreatic β-cells. Together, these data suggest that loss of miR-483 increases ALDH1A3 expression, which may contribute to impaired β-cell identity and function under metabolic stress.

### Single-cell RNA sequencing reveals that loss of miR-483 increases β-cell heterogeneity and acquisition of α-cell–like features

To determine how β-cells are altered upon loss of miR-483, we performed scRNA-seq on islet cells isolated from HFD/STZ-treated miR483^+^/^+^ and miR483^−^/^−^ mice on day 10 post-STZ. A total of 11 071 islet cells were profiled (5753 from miR483^+^/^+^ and 5318 from miR483^−^/^−^). Unsupervised graph-based clustering combined with marker gene analysis identified all major endocrine cell types, which were similarly represented in both groups, with α-cells and β-cells comprising more than 80% of all cells.

We selected α- and β-cell populations for further analysis (Fig. 3A). Based on GFP, INS2, and GCG expression, β-cells segregated into 5 clusters (clusters 1-5), whereas α-cells into 4 clusters (clusters 6-9) (Fig. 3B-3D; Table S1) (27). Notably, GFP expression was not only detected across all β-cell clusters but also observed in the GCG-positive cluster 8. Cluster 8 exhibited a mixed transcriptional profile, with expression of β-cell–associated features together with increased expression of α-cell identity genes (Arx, MafB, Irx1, Irx2), as well as stress- or dedifferentiation-associated markers (Oat, Id1, Id3) (Fig. 3E; Table S2) (27). These findings suggest that cluster 8 represents a transitional population exhibiting both β-cell–associated and α-cell–like features. Gene Ontology enrichment analysis of DEGs in cluster 8 revealed significant upregulation of pathways associated with cellular signaling, loss of cell identity, apoptotic signaling, and actin filament organization, including cell surface receptor signaling pathway, negative regulation of cell development, and regulation of apoptotic signaling pathway (Fig. 3F). Together, these findings indicate that loss of miR-483 under metabolic stress is associated with increased β-cell heterogeneity and the emergence of a distinct population exhibiting α-cell–like and stress-associated transcriptional features, consistent with increased β-cell plasticity and identity loss.

**Figure 3:**
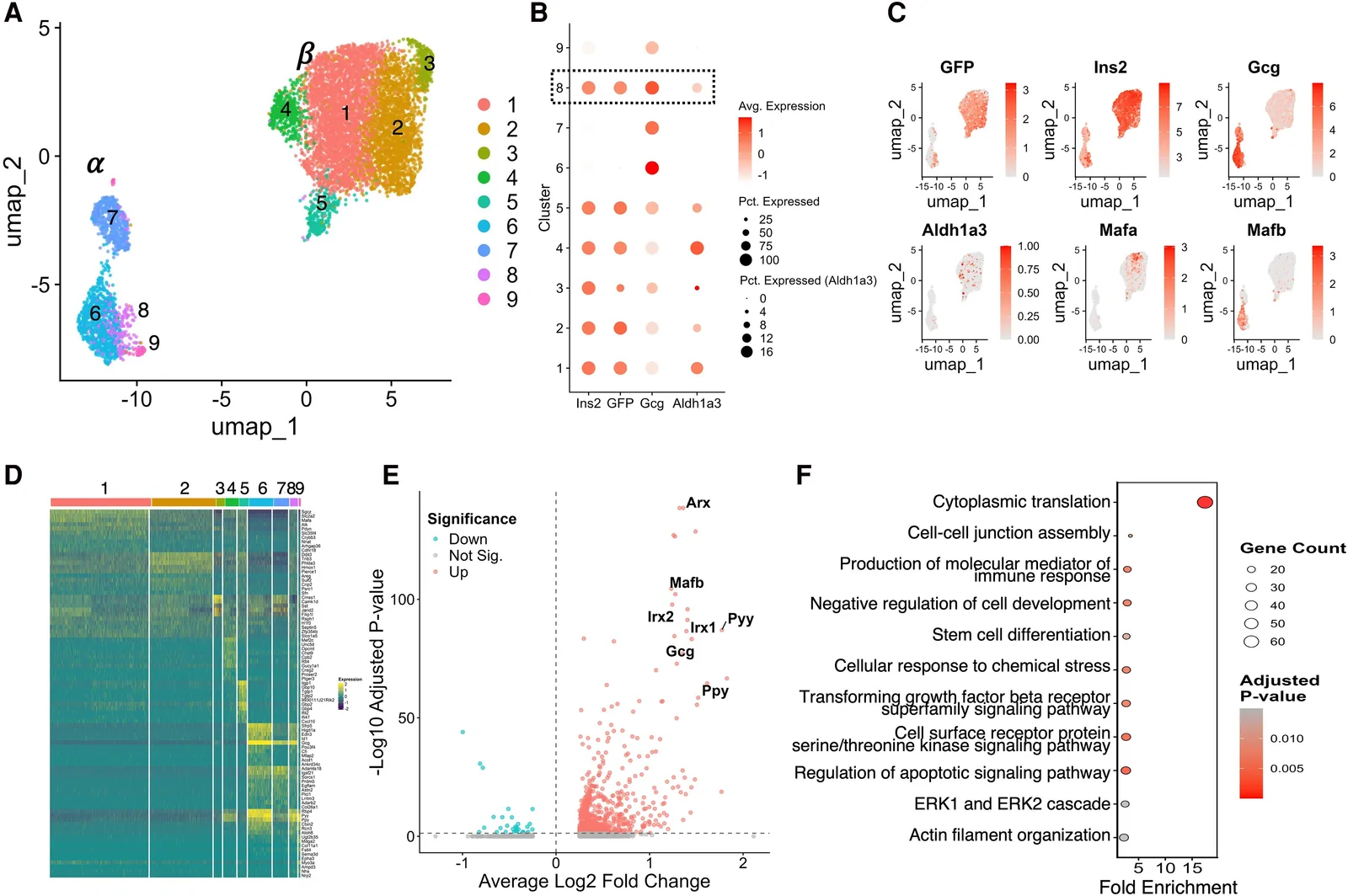
scRNA-seq profiling of GFP-lineage-traced cells from miR483^+^/^+^ and miR483^−^/^−^ islets. (A) UMAP representation showing 9 distinct clusters of α- and β-cells derived from miR483^+^/^+^ and miR483^−^/^−^ islets on day 10 post-STZ. Clusters are numbered and color coded as indicated. (B) Dot plot illustrating expression patterns of lineage and endocrine identity markers (GFP, INS2, GCG, ALDH1A3). Cluster 8 is characterized by co-expression of GFP, INS, and GCG. (C) UMAP feature plots displaying expression of GFP, INS2, GCG, ALDH1A3, MafA, and MafB. (D) Heatmap of top 10 differentially expressed genes (DEGs) of each cluster. The top annotation bar denotes cluster identities; corresponding gene lists are provided in Table S1 (27). (E) Volcano plot highlighting upregulated and downregulated DEGs in cluster 8. The complete DEG lists are included in Table S2 (27). (F) Significantly enriched genes (DEGs) in cluster 8 were subjected to Gene Ontology (GO) enrichment analysis of Biological Process (BP) terms. The significantly enriched terms are shown (adjusted *P* < .05). The x-axis denotes gene ratio (count/set size) and dot size indicates gene counts; adjusted *P* values are provided as indicated in the figure.

When comparing miR483^−^/^−^ and control miR483^+^/^+^ islet cells, UMAP analysis revealed an expansion of cluster 8 in miR483^−^/^−^ compared with controls (59% vs 41%, respectively) (Fig. 4A and 4B). Among β-cell populations, cluster 1 increased, whereas cluster 2 was reduced in miR483^−^/^−^ islets, suggesting alterations in β-cell transcriptional states (Fig. 4B). Differential gene expression analysis confirmed significant upregulation of ALDH1A3 in miR483^−^/^−^ β-cells (Fig. 4C), consistent with the increased ALDH1A3 protein expression observed by immunostaining and Western blot analysis (Fig. 2). Aldehyde dehydrogenase 1A3 expression was detected in clusters 1 to 5 and 8, with marked enrichment in cluster 1 (Fig. 3B and 3C; Table S1) (27), further supporting alterations in β-cell identity following miR-483 loss.

**Figure 4:**
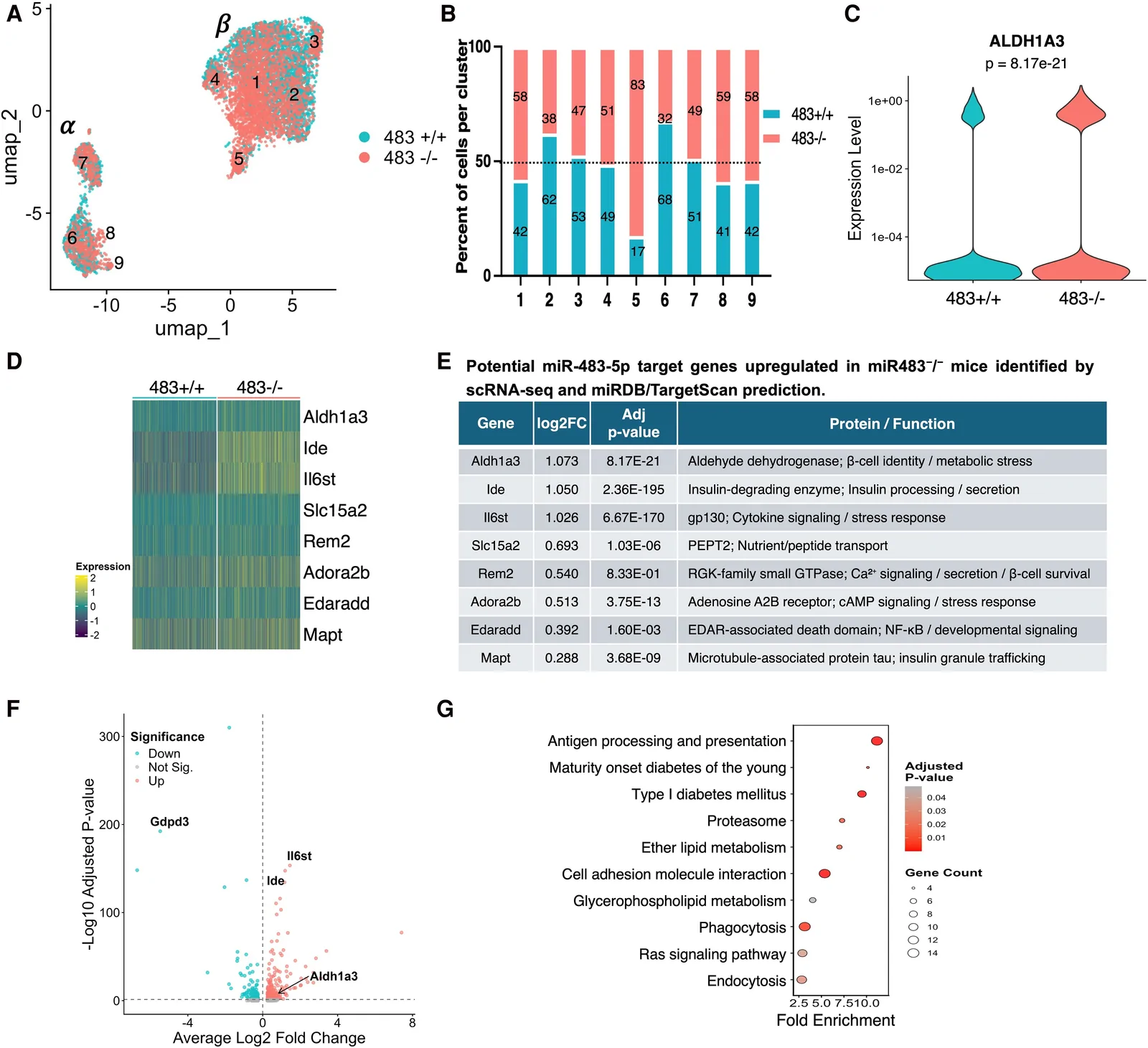
scRNA-seq reveals increased β-cell heterogeneity and acquisition of α-cell–like features following miR-483 deficiency. (A) UMAP visualization separated by genotype (483^+^/^+^ vs 483^−^/^−^). Each dot represents a single cell and is colored by sample identity. (B) Relative abundance of miR483^+^/^+^ and miR483^−^/^−^ cells across individual cell cluster. (C). Violin plots illustrating increased ALDH1A3 expression in miR483^−^/^−^ cells. (D) Heatmap showing the expression of predicted miR-483 target genes identified using miRDB and TargetScan. (E) List of 8 candidate target genes of miR-483 that were significantly upregulated in miR483^−^/^−^ cells. (F) Volcano plot displaying upregulated and downregulated DEGs between miR483^+^/^+^ and miR483^−^/^−^ cells. The complete DEG lists are included in Table S3 (27). (G) Enriched KEGG pathways of DEGs between miR483^+^/^+^ and miR483^−^/^−^ cells. The x-axis denotes log2 fold enrichment and dot size indicates gene count; adjusted *P* values are provided as indicated in the figure.

Because miRNAs can regulate multiple target genes, we further examined additional potential miR-483 targets. In addition to the validated direct target Aldh1a3, 7 additional potential targets were identified using miRDB and TargetScan prediction programs and were significantly upregulated in the combined analysis of all clusters in miR483^−^/^−^ compared with miR483^+^/^+^ islet cells (Fig. 4D and 4E). Several of these genes, including Il6st and Ide, have previously been implicated in β-cell function or cellular stress responses, suggesting that dysregulation of multiple miR-483-responsive genes may contribute to β-cell dysfunction under metabolic stress.

In addition, numerous DEGs in miR483^−^/^−^ islets encoded proteins involved in mitochondrial function and oxidative stress (Fig. 4F; Table S3) (27). Kyoto Encyclopedia of Genes and Genomes enrichment analysis of DEGs further revealed significant enrichment of several pathways associated with β-cell dysfunction and cellular stress, including maturity onset diabetes of the young, Ras signaling, cell adhesion molecule interactions, and lipid metabolism pathways (Fig. 4G). Notably, cluster 5 showed a marked increase in miR483^−^/^−^ islets, rising from 17% of cells in miR483^+^/^+^ control to 83% in miR483^−^/^−^ islets (Fig. 4B). Gene Ontology analysis of this cluster showed strong enrichment of innate immune response pathways, suggesting that loss of miR-483 may be associated with an enhanced inflammatory state within the islet. Together, these findings demonstrate that loss of miR-483 is associated with β-cell heterogeneity, dysregulation of the miR-483–ALDH1A3 axis, mitochondrial and stress-related transcriptional changes, and the emergence of β-cell populations exhibiting α-cell–like features under metabolic stress.

### miR-483 deficiency increases glucagon production and MafB-positive β-cell features under high-fat diet/streptozotocin-induced stress

To determine whether miR-483 deficiency promotes β-cell loss and acquisition of α-cell–like features under metabolic stress, we examined glucagon expression in HFD/STZ-treated mice. Following STZ treatment, miR483^−^/^−^ mice exhibited substantially higher fasting plasma glucagon levels than miR483^+^/^+^ control mice on days 7, 14, and 21 (Fig. 5A). Following glucose administration, plasma glucagon levels decreased in miR483^−^/^−^ mice but remained significantly higher than those in miR483^+^/^+^ control mice at 15 minutes (Fig. 5B). These findings indicate that miR-483 deficiency is associated with increased basal glucagon levels and impaired glucose-mediated suppression of glucagon secretion. Consistent with these findings, islet glucagon content was increased nearly 2-fold in miR483^−^/^−^ mice compared with miR483^+^/^+^ controls on day 15 (Fig. 5C).

**Figure 5:**
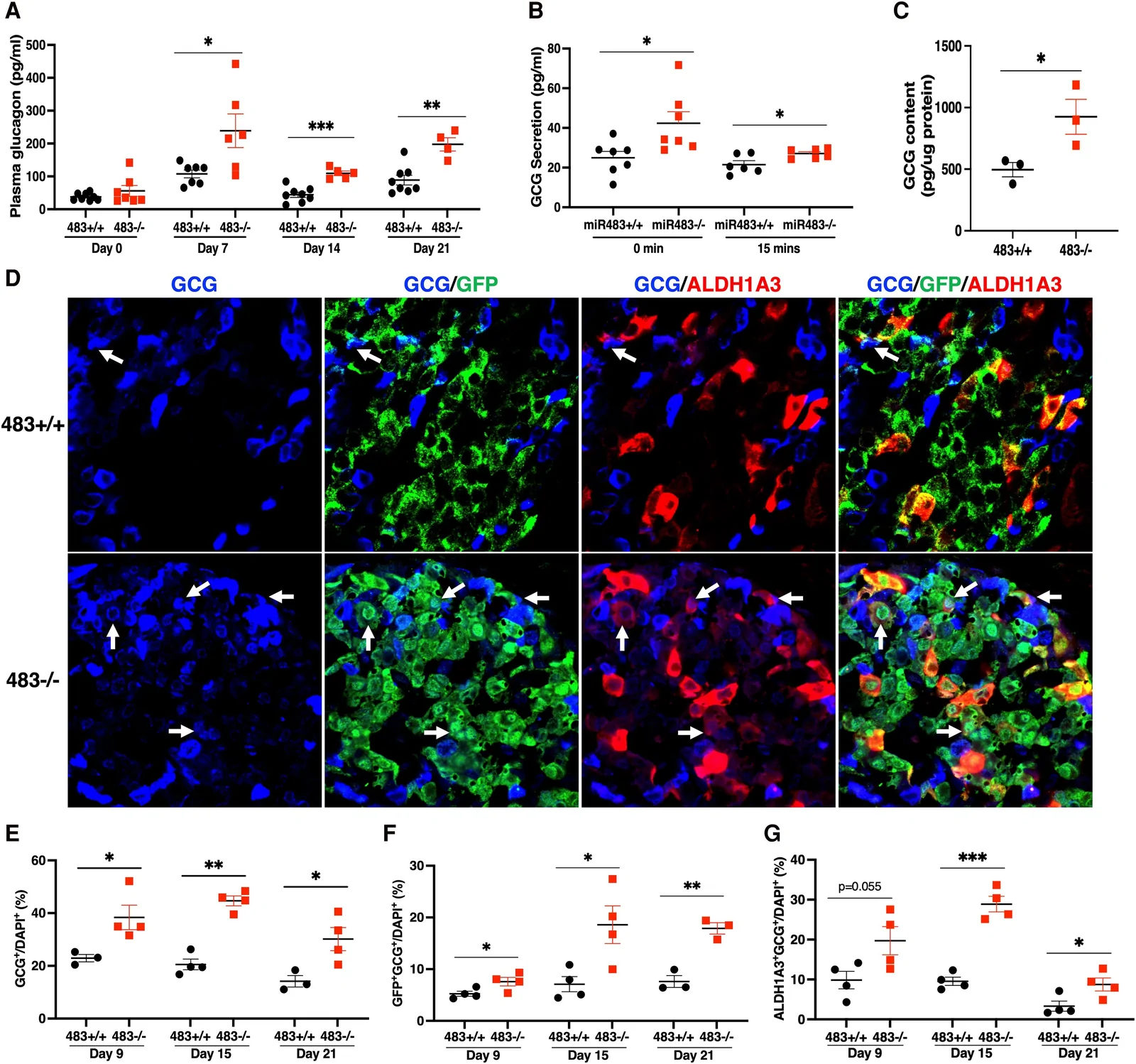
miR-483 deficiency increases glucagon production and GFP-labeled glucagon-positive cells under HFD/STZ-induced metabolic stress. Mice were fed HFD for 12 weeks and then treated with STZ (50 mg/kg × 5 days). (A) Plasma glucagon on days 7, 14, and 21 post-STZ in miR483^−^/^−^ and miR483^+^/^+^ mice. Plasma was collected after a 6-hour fast. (B) Plasma glucagon levels measured at baseline (0 minutes) and 15 minutes after intraperitoneal glucose administration (1 g/kg body weight) on day 21 post-STZ following a 6-hour fast (n = 6-7 per group). (C) Glucagon content in isolated islets on day 10 post-STZ, normalized to total protein content (n = 3 per group). (D) Immunofluorescence images of pancreatic sections showing glucagon (GCG), GFP, and ALDH1A3. White arrowheads indicate GCG^+^GFP^+^ALDH1A3^+^ cells. (E-G) Quantification of total GCG^+^ (E), GFP^+^GCG^+^(F), and ALDH1A3^+^GCG^+^ (G) cells, showing significant increase in miR483^−^/^−^ mice. Data are mean ± SEM (n = 4-8). *P < .05, **P < .01, ***P < .001 vs miR483^+^/^+^.

Immunostaining of pancreatic sections further revealed an expanded glucagon^+^ cell population in miR483^−^/^−^ mice, which was approximately 2-fold higher than that in control mice on day 15 post-STZ (Fig. 5D and 5E). Lineage-tracing analysis showed that the proportion of GFP^+^ cells co-expressing glucagon was increased in miR483^−^/^−^ mice compared with controls (20% vs 10%, respectively, on day 15 post-STZ; Fig. 5F). Similarly, the proportion of ALDH1A3^+^ cells co-expressing glucagon was greater in miR483^−^/^−^ mice than in controls (30% vs 10%, respectively, on day 15 post-STZ; Fig. 5G). Together, these findings indicate that loss of miR-483 deficiency promotes loss of β-cell identity and acquisition of α-cell–like features under metabolic stress.

Lineage tracing was further used to assess expression of MafB, a transcription factor characteristic of α-cells that is normally expressed at low levels in mature β-cells. Immunostaining revealed markedly increased MafB expression in miR483^−^/^−^ islets compared with miR483^+^/^+^ control islets (Fig. 6A and 6B). A subset of GFP^+^ cells aberrantly co-expressed MafB, indicating acquisition of α-cell–like features within the GFP^+^ insulin-expressing lineage. Quantitative analysis confirmed a significant increase in GFP^+^MafB^+^ cells in miR483^−^/^−^ islets compared with miR483^+^/^+^ controls (Fig. 6C). Together, these findings support increased β-cell identity loss and acquisition of α-cell–like features following miR-483 deficiency.

**Figure 6:**
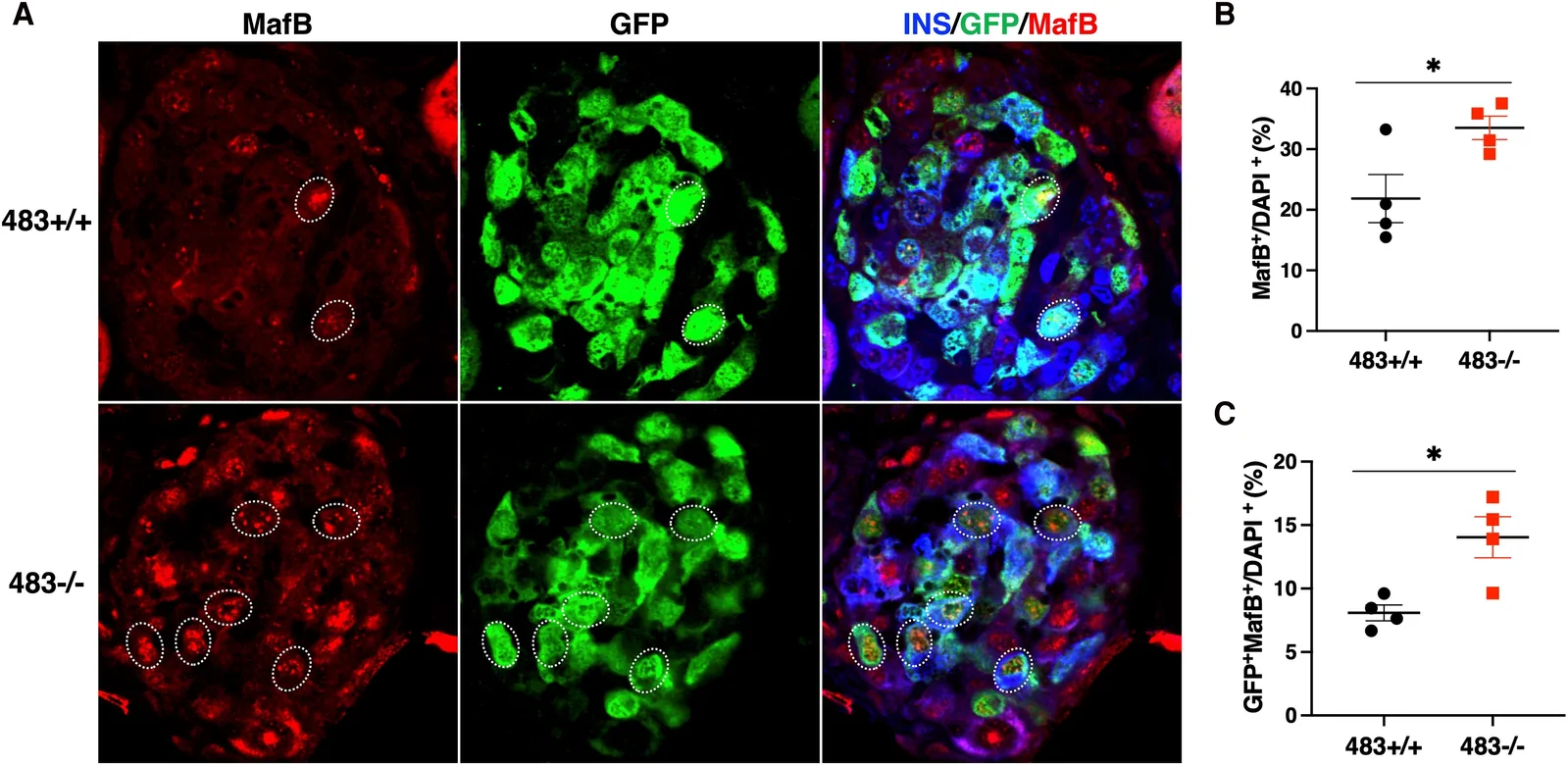
miR-483 deficiency increases MafB expression and MafB-positive β-cell features under HFD/STZ-induced metabolic stress. (A) Representative immunofluorescence of pancreas sections stained for insulin (INS), GFP, and MafB. (B-C). Quantification of MafB^+^ (B) and MafB^+^GFP^+^ (C) cells on day 15 post-STZ. White circles indicate MafB⁺GFP⁺ cells. Data are mean ± SEM (n = 4). *P < .05 vs miR483^+^/^+^.

### Loss of miR-483 induces mitochondria fragmentation and dysfunction

Our transcriptomic analysis revealed that miR-483 deficiency alters the blood lipid profile and increases the expression of multiple mitochondrial metabolic enzymes, suggesting that loss of miR-483 may impair mitochondrial homeostasis. To directly assess mitochondrial integrity, we examined mitochondrial morphology using Tom20 immunostaining. In control β-cells, mitochondria displayed a well-organized, reticular tubular network extending throughout the cytoplasm (Fig. 7A and 7B). In contrast, miR483^−^/^−^ β-cells showed a marked shift toward perinuclear mitochondrial accumulation accompanied by pronounced fragmentation and dense cluster formation, features consistent with mitochondrial stress or dysfunction. Intracellular ROS levels were quantified using DCFH-DA oxidation. miR483^−^/^−^ islets exhibited significantly elevated ROS compared with controls (Fig. 7C), supporting the notion that loss of miR-483 increases oxidative stress.

**Figure 7:**
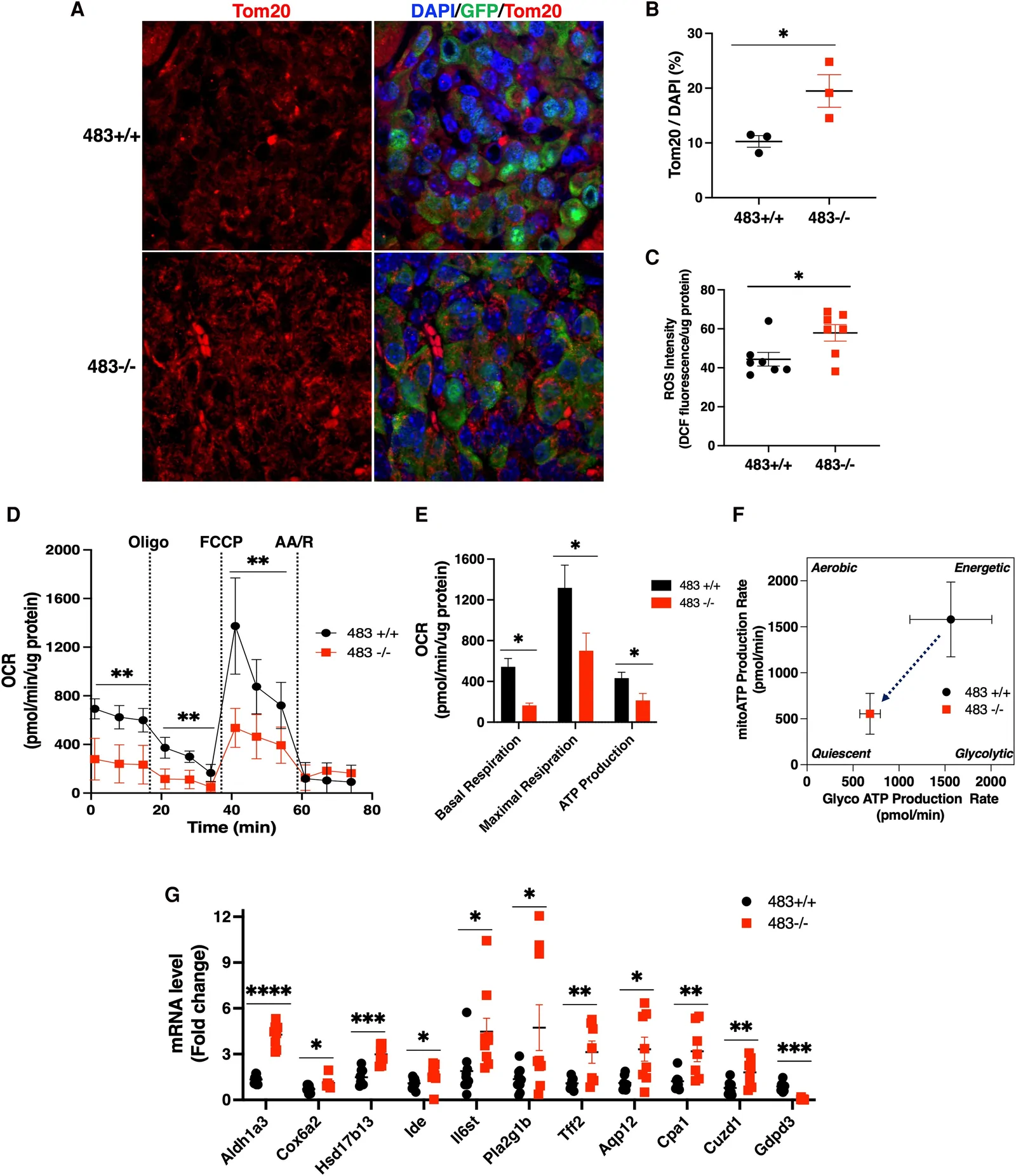
miR-483 loss promotes mitochondrial fragmentation and dysfunction. (A) Immunofluorescence of pancreatic β-cells showing mitochondria (Tom20) and GFP. (B) Quantification of Tom20^+^ cells on day 15 post-STZ. *P < .05. (C) Intracellular ROS levels in isolated islets measured by DCFH-DA assay. (D) Oxygen consumption rates (OCRs) of 20 dispersed islets measured by XFp Seahorse Analyzer following oligomycin (1.5 μM), FCCP (1 μM), and antimycin/rotenone (0.5 μM each). (E) Quantification of basal respiration, maximal respiration, and ATP production. (F) miR-483 deficiency shifted islets from the energetically active state in control mice toward a quiescent state. (G) Expression of mitochondria-related genes assessed by qPCR. Data are mean ± SD of 3 independent experiments. *P < .05, **P < .01, ***P < .001, ****P < .0001 vs miR483^+^/^+^.

To further evaluate mitochondrial functional integrity, we measured the OCR in isolated islets 10 days after STZ injection. miR-483 deficiency resulted in substantial impairments in mitochondrial respiration, with basal and maximal OCR reduced by approximately 30% and 50%, respectively, compared with control islets (Fig. 7D and 7E). Consistent with these respiratory defects, ATP production was markedly decreased in miR483^−^/^−^ islets (Fig. 7E). Moreover, miR-483 deficiency shifted islets from the energetically active state observed in control mice toward a metabolically compromised, quiescent state (Fig. 7F), indicating severe mitochondrial dysfunction.

In parallel, RNA-seq analysis has identified a distinct set of metabolic and mitochondrial genes whose expression was altered in the absence of miR-483. To validate these findings, we performed RT-qPCR on islets isolated from miR483^−^/^−^ and miR483^+^/^+^ mice. As expected, Aldh1a3 was robustly upregulated, consistent with impaired mitochondrial function and increased oxidative stress (Fig. 7G). The 2 predicted miR-483 targets, Ide and Il6st, were also significantly increased in miR483^−^/^−^ islets. Genes involved in mitochondrial metabolism and β-oxidation, including Cox6a2 and Hsd17b13, as well as genes associated with cellular stress responses, including Pla2g1b and Tff2, were also significantly upregulated in miR483^−^/^−^ islets (Fig. 7G). In addition, several genes encoding secretory and digestive enzymes, including Aqp12, Cpa1, and Cuzd1, were elevated, consistent with loss of β-cell identity and acquisition of acinar/exocrine cell characteristics. In contrast, Gdpd3, a glycerophosphodiester phosphodiesterase involved in lipid metabolic flux and membrane remodeling, was markedly reduced in miR483^−^/^−^ islets. Collectively, these transcriptional alterations are consistent with the observed defects in mitochondrial morphology and respiration and support a role for miR-483 in maintaining mitochondrial integrity and metabolic function in β-cells.

## Discussion

In this study, we identify miR-483 as an important regulator of β-cell identity and stress adaptation. Although miR-483 deficiency does not produce an overt metabolic phenotype under basal conditions (20), its loss increases β-cell susceptibility to metabolic stress induced by HFD/STZ treatment. Using lineage-tracing approaches, scRNA-seq, and functional metabolic assays, we show that β-cell–specific deletion of miR-483 is associated with increased ALDH1A3 expression, greater β-cell heterogeneity, loss of β-cell identity, and the emergence of β-cell populations with α-cell–like features following metabolic stress. These findings build upon and extend previous work demonstrating that miRNAs are important regulators of β-cell identity and stress resistance (12, 28).

Aldehyde dehydrogenase 1A3 is a well-established marker of failed or dedifferentiated β-cells in both rodent and human diabetes. Numerous studies have shown that ALDH1A3 expression rises sharply in dysfunctional β-cells exposed to glucolipotoxicity, cytokines, or metabolic stress (7, 9). In our previous study, we found that miR-483 deletion increased *Aldh1a3* expression following either HFD feeding or STZ treatment, suggesting that miR-483 regulates ALDH1A3 in response to metabolic stress or β-cell injury (20). Importantly, ALDH1A3 induction has been reported to occur early during β-cell stress, while suppression of ALDH1A3 can promote β-cell recovery, allowing dedifferentiated cells to reacquire mature β-cell characteristics and restore insulin secretory capacity (10). Our lineage-tracing analysis showed that ALDH1A3^+^ cells were detected within the GFP^+^ insulin-expressing lineage, supporting their association with the β-cell lineage. Together with our previous evidence that miR-483 directly targets Aldh1a3, these findings support the miR483–ALDH1A3 regulatory axis as an important regulator of β-cell identity under metabolic stress.

Multiple lines of evidence support the concept that stressed β-cells can acquire α-like molecular features. Genetic studies demonstrate that loss of key β-cell transcription factors, such as FOXO1, PDX1, and NKX2.2, downregulates β-cell identity programs while activating α-cell transcriptional networks, promoting β-cell phenotypic conversion (2, 29, 30). Human islet studies similarly suggest that β-to-α conversion occurs in T2D and correlates with hyperglucagonemia (31). Consistent with these reports, our single-cell analysis identified distinct GCG^+^ populations (cluster 8) in miR-483-deficient islets that retained β-cell–associated features while expressing α-cell markers, suggesting acquisition of an α-like phenotype. Increased MafB expression in GFP^+^ cells further supports this phenotypic shift, as MafB is normally expressed in adult α-cells and can be reactivated during β-cell identity loss (32). Cluster 8 also showed increased expression of α-cell identity genes, including Arx, Irx1, and Irx2, together with stress- and dedifferentiation-associated markers, such as Oat, Id1 and Id3 (33-35). In addition, increased Ppy and Pyy expression is consistent with previous studies showing the emergence of PP-cell–like programs during β-cell instability (36-38). Together, these findings suggest that miR-483 helps maintain β-cell identity and limits the acquisition of α-like and other endocrine-cell features under metabolic stress.

A major finding of this study is that loss of miR-483 disrupts mitochondrial morphology and function in β-cells, as evidenced by mitochondrial fragmentation, reduced oxygen consumption, diminished ATP production, and elevated ROS (39, 40). Importantly, recent studies demonstrate that mitochondrial metabolic state can regulate β-cell identity and fate (41, 42). Disruption of mitochondrial quality-control pathways promotes loss of β-cell maturity and identity, whereas inhibition of the mitochondrial integrated stress response can restore β-cell identity (43). Similarly, β-cell–specific deletion of Cyb5r3, a regulator of mitochondrial electron transport and redox homeostasis, impairs mitochondrial function and reduces β-cell differentiation markers (44). These findings support a role for mitochondrial dysfunction in promoting loss of mature β-cell characteristics.

Our finding that miR-483 deficiency increases ALDH1A3, a direct miR-483 target, provides a potential link between miR-483 loss, metabolic/mitochondrial dysfunction, and β-cell identity. Aldehyde dehydrogenase 1A3 may further promote oxidative and metabolic stress through altered retinoid and lipid metabolism (10). Consistent with this model, our transcriptomic analyses revealed altered expression of genes involved in mitochondrial and lipid metabolism (eg, *Cox6a2*, *Hsd17b13*), stress-response regulators (*Ide*, *Il6st*) that are predicted potential direct targets of miR-483, and genes associated with acinar/exocrine cell characteristics (*Cpa1*, *Aqp12, Cuzd1*). Together, these findings support a model in which the miR-483–ALDH1A3 axis, potentially involving additional targets, such as Ide and Il6st, links metabolic stress and mitochondrial dysfunction to loss of β-cell identity.

A limitation of this study is the use of constitutive Ins1-Cre lineage tracing, which lacks temporal control of β-cell labeling. Thus, some YFP^+^GCG^+^ cells may have acquired Ins1 promoter activity during disease progression rather than originated from pre-existing β-cells. Therefore, these findings provide supportive but not definitive evidence of β-cell-to-α-cell transdifferentiation. Nevertheless, β-cell identity loss is supported by increased ALDH1A3 and MafB, reduced β-cell identity genes, altered glucagon production, and a transitional β-cell population identified by scRNA-seq. Future studies using inducible Ins1-CreERT2 lineage tracing will be needed to definitively establish the origin and fate of these transitional cells.

Together, these data support a model in which miR-483 contributes to the maintenance of β-cell identity and mitochondrial function during metabolic stress. Loss of miR-483 is associated with increased ALDH1A3 expression, impaired β-cell identity, acquisition of alternative endocrine features, and mitochondrial dysfunction, contributing to β-cell dysfunction and hyperglucagonemia. Although the results support ALDH1A3 as a downstream effector of miR-483, whether miR-483 restoration or ALDH1A3 inhibition can rescue β-cell identity loss remains to be established. Nevertheless, our findings identify the miR-483–ALDH1A3 axis as a potential mechanism linking metabolic stress to loss of β-cell identity and function.

## Data Availability

Original data generated and analyzed during this study are included in this published article or in the data repository listed in Reference (27): https://doi.org/10.6084/m9.figshare.32148322.
